# A Fuzzy-Match Search Engine for Physician Directories

**DOI:** 10.2196/medinform.3463

**Published:** 2014-11-04

**Authors:** Majid Rastegar-Mojarad, Christopher Kadolph, Zhan Ye, Daniel Wall, Narayana Murali, Simon Lin

**Affiliations:** ^1^Marshfield Clinic Research FoundationBiomedical Informatics Research CenterMarshfield, WIUnited States; ^2^Marshfield ClinicDepartment of NephrologyMarshfield, WIUnited States; ^3^The Research Institute at Nationwide Children's HospitalColumbus, OHUnited States

**Keywords:** Fuzzy-Match, Levenshtein Distance, Physician Name, Physician Directory

## Abstract

**Background:**

A search engine to find physicians’ information is a basic but crucial function of a health care provider’s website. Inefficient search engines, which return no results or incorrect results, can lead to patient frustration and potential customer loss. A search engine that can handle misspellings and spelling variations of names is needed, as the United States (US) has culturally, racially, and ethnically diverse names.

**Objective:**

The Marshfield Clinic website provides a search engine for users to search for physicians’ names. The current search engine provides an auto-completion function, but it requires an exact match. We observed that 26% of all searches yielded no results. The goal was to design a fuzzy-match algorithm to aid users in finding physicians easier and faster.

**Methods:**

Instead of an exact match search, we used a fuzzy algorithm to find similar matches for searched terms. In the algorithm, we solved three types of search engine failures: “Typographic”, “Phonetic spelling variation”, and “Nickname”. To solve these mismatches, we used a customized Levenshtein distance calculation that incorporated Soundex coding and a lookup table of nicknames derived from US census data.

**Results:**

Using the “Challenge Data Set of Marshfield Physician Names,” we evaluated the accuracy of fuzzy-match engine–top ten (90%) and compared it with exact match (0%), Soundex (24%), Levenshtein distance (59%), and fuzzy-match engine–top one (71%).

**Conclusions:**

We designed, created a reference implementation, and evaluated a fuzzy-match search engine for physician directories. The open-source code is available at the codeplex website and a reference implementation is available for demonstration at the datamarsh website.

##  Introduction

A primary functionality of the website of a physician group practice is a search engine where patients can enter a physician's name and find more information about the physician’s practice, credentials, and appointment phone number. Name-based searching seems to be a simple task, but various types of spelling mismatches caused by typographical errors, phonetic spelling variations, and nicknames can make the task difficult. Failure to find a physician on the provider’s website can create a frustrating experience for the patient and potential loss of business for the provider.

We surveyed the websites of the ten largest medical groups [1], and found none of them allowed mismatched characters in the entered name. 7 of the 10 search engines allowed autocomplete, which tries to finish the rest of the characters based on what has already been typed. However, current implementations of auto-complete require 100% match in the already typed fragments, any mismatches will end up with no results. The Google search engine does allow fuzzy-match, but it is not specific to the physician directory on a provider’s website. Consequently, a general Google search of a physician’s name might lead to websites other than the provider’s. As such, the Google search does not provide an integrated patient experience at the provider’s website. Currently, there are no open-source solutions of a fuzzy-match search engine for physician directories.

To improve upon current and severely limited provider search engines, we conducted a heuristic analysis of the search log. A common mismatch can be caused by typographical errors. For example, “Smith” is entered as “Smitj”, because the “j” key is adjacent to the “h” key. As more people are searching websites using smaller touch-screen devices such as smartphones, typographical errors resulting from adjacent keys are becoming more common. Levenshtein distance based methods, as previously used in matching drug names and chemical names [2,3], can be effective in correcting this type of error. Levenshtein distance is a measure of the similarity between two strings. The distance is the number of deletions, insertions, or substitutions required to transform one string to the other. For instance, the Levenshtein distance between “Smith” and “Smitj” is one, whereas an exact match results in a distance of zero.

Another type of mismatch is caused by phonetic variations in names. For instance, “Smith” and “Smyth” are pronounced the same but spelled differently. Sound-based encoding methods such as Soundex and Metaphone were designed to solve the phonetic variation in names. In 1918, Robert Russell developed the first Soundex system and subsequently, several implementations were devised. Soundex encodes [4] names based on their sound, so that names with close pronunciation get the same code. For example, both “Smith” and “Smyth” are coded as “S530”. One problem with Soundex is that it returns many approximate matches, with most being far from the searched-for name [5]. Beidar and Morse [5] developed the Beider-Morse Phonetic Matching system for decreasing the number of approximate matches by removing irrelevant ones. Lawrence Phillips upgraded the Soundex system in 1990 and developed Metaphone [6], which produces more accurate encoding of names that sound similar. Further development of Double Metaphone [6] enabled two codes for a single name to account for different kinds of spelling variations. Double Metaphone also improved the match of non-English names. However, implementations of Soundex or Metaphone are usually outside of the aforementioned Levenshtein distance framework.

A third type of variation is caused by nicknames. For instance, “Bill” might exist in the directory as “William.” Since “Bill” and “William” do not sound, nor are spelled alike, nicknames pose another challenge for name searches. Nicknames cannot be resolved by distance-based match or sound-based match. None of the search engines at the ten largest medical groups had a good solution for nicknames. We proposed to use a nickname lookup table [7] derived from the United States (US) census data to solve this problem, where we also incorporated it in the Levenshtein distance framework.

In the medical informatics literature, the approximate match of patient names has been studied extensively. Both phonetic name matching and Levenshtein distance based methods were reported [8,9]. Peter Christen [10] presented a comprehensive review on the name matching algorithms; however, there have been no reports of an integrated solution that simultaneously addresses all three kinds of mismatches.

Marshfield Clinic has more than 800 providers with diverse first and last names. A fast and effective “Find a doctor” engine is critical to the business operation. From the log file of the “Find a doctor” webpage at Marshfield Clinic, we observed that 26% of the 9072 searches in July 2013 yielded no results. To aid patients in finding the wanted provider easier and faster, we suggest a list of providers’ name that are similar to the search term. As a patient enters the name of the desired physician, our system provides a list of suggestions that helps the user, even if they do not know the correct spelling of the wanted physician’s name. Unlike most available systems, our system applies approximate search instead of exact match search for finding similar names. This article presents an open-source solution, demonstrates the implementation, and evaluates the effectiveness of a fuzzy search engine for physician directories. The novelty in our system is that it is the first open-source search engine for physician directories that solves all three kinds of spelling mismatches: typographical errors, phonetic variations, and nicknames.

## Methods

In our application, it was imperative to find the closest physician’s name in the directory to the entered search term. First, we performed some preprocessing steps. We removed common prefixes and suffixes in the string, such as Dr, MD, FACS, etc. Then, to solve all three kinds of mismatches in a unified framework, we customized the Levenshtein distance method. Refer to Textbox 1. for the assigned cost for each operation.

**Cost of operation.**

1. Cost of deletion is:

I. 4 if the letter is ‘a’, ‘e’, ‘i’, ‘o’, or ‘u’

II. 4 if the letter is the same as the previous letter (repetitive letters)

III. otherwise 5

2. Cost of substitution is:

I. 3 if both letters have a similar sound. Here, we used Soundex to determine whether two letters have the same sound. For example, we assumed that ‘d’ and ‘t’ have the same sound, because they have the same code in Soundex.

II. 3 if they are adjacent on keyboard. We took eight surrounding keys for each character and assigned them with lower penalties to accommodate typographical errors.

III. otherwise 4

Additionally, we used the nickname lookup table to expand the match to the physician directory. Each nickname is assigned with a matching likelihood. For instance, “William” has a 0.9 chance of being called “Bill” and 0.45 chance of being called “Will”. We also incorporated the probability in the final matching score.

To evaluate the performance of the method, we chose 100 recently searched terms from the Marshfield Clinic website’s current search engine (uses exact match approach) log file, which did not return any results. Using human intelligence, we identified the correct physicians name in the Marshfield Clinic directory for 68 of the searched terms. We call this gold-standard data set the “Challenge Data Set of Marshfield Physician Names”. Ten examples in this data set are shown in Table 1.

**Table 1:** Example data in the “Challenge Data Set of Marshfield Physician Names”.

Search term entered by patient	Actual name in the directory
alvarex	Maria Alvarez
carrie tull	Carie Tull
Ceasar gonzaga	Caesar Gonzaga
phillip zickerman	Philip Zickerman
reinhardt	Richard Reinhart
roedrick koehler	Roderick Koehler
rousch	Stephen Roush
scott erickwon	Scott Erickson
STEVEN TOOTHACKER	Stephen Toothaker
tim swan	Timothy Swan

To compare diversity of the names of US physicians versus general US population, a list of 1,048,576 physician names was obtained from the National Provider Identifier Registry of 2013 [11]. The names of the general US population were obtained from the website of the US Census Bureau [12]. Because the 1990 census is the latest one containing statistics with both first and last names, we used it in this study.

##  Results

**Figure 1:**
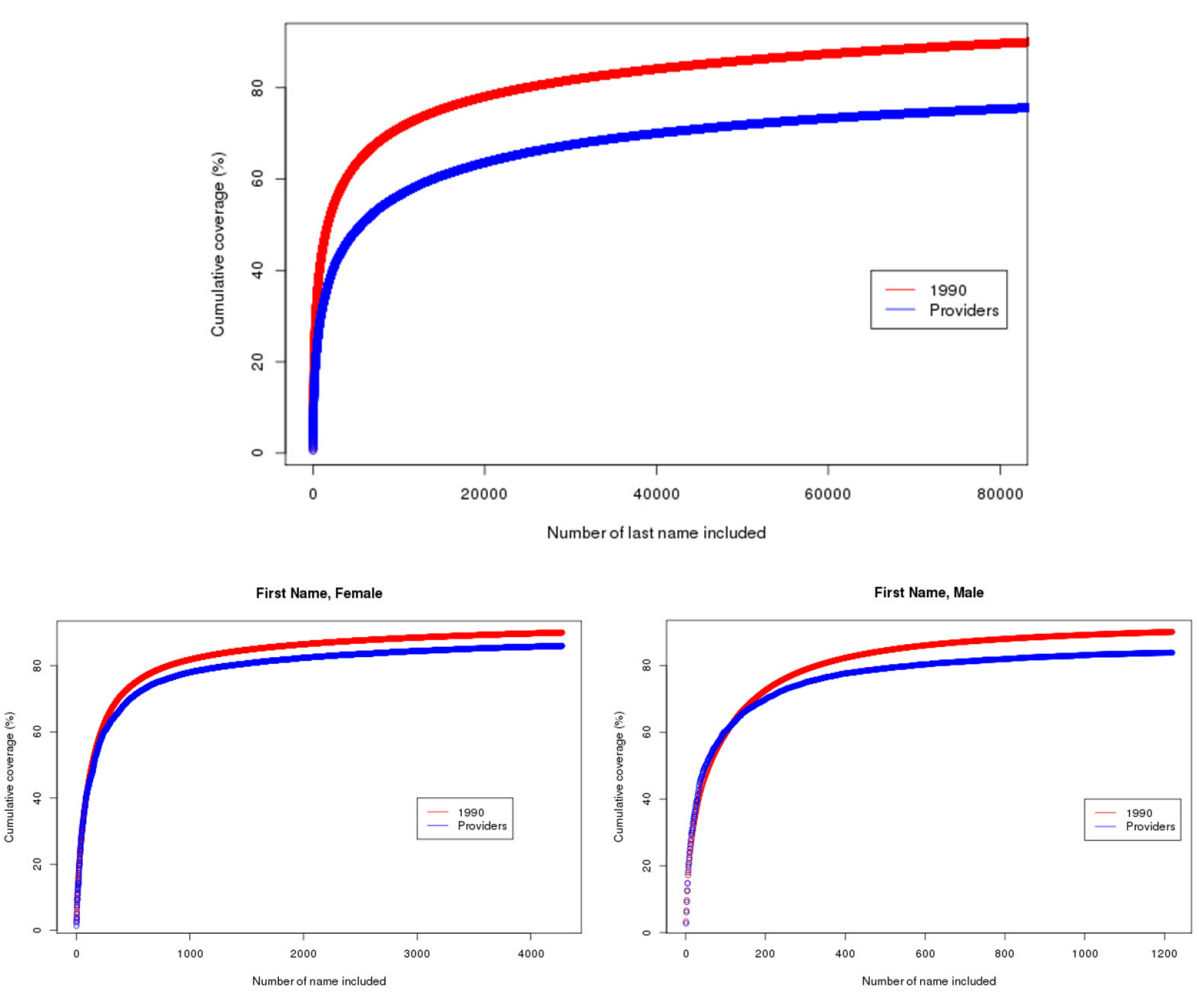
Physician’s first name and last name, comparing with general US population.

It is important to note that physician names are more diverse than those of the general US population. By comparing the nationwide physician names listed in the National Provider Identifier registry with the general US population, we confirmed that the physician names are less common than names in the general US population (Figure 1). For instance, to cover 70% of the last names, 9028 names need to be included for the general US population, whereas 40,014 names need to be included for physician names. The same is true for first names, but to a lesser extent (Figure 1). Consequently, less common names can be more challenging to spell correctly. To assess the statistical significance, we utilized two sample Kolmogorov-Smirnov (K-S) tests on the two cumulative distributions from each of the three graphs in Figure 1. The results show P values <.001, which indicates there are significant differences between the two distributions of the cumulative coverage of physician last names, male first names, and female first names, respectively.

Less common names, combined with phonetic variations, nicknames, and typographical errors, pose challenges to search engines at a group practice provider’s website. We researched the “Find a Doctor” webpages at the top 10 medical groups in the United States (Table 2). None of the websites allowed fuzzy-match of physicians’ names. While 7 out of 10 websites have the autocompletion feature, none allow any mismatches in the name search query.

**Table 2:** “Find a doctor” search engines at top ten medical groups in the United States [3].

Medical group	Headquarters	Offices	Physicians	Auto completion
Kaiser Permanente Medical Group	Santa Clara, CA	484	7842	No
Cleveland Clinic	Cleveland, OH	173	1472	Yes
Henry Ford Medical Group	Detroit, MI	218	1224	Yes
IU Health Physicians	Indianapolis, IN	267	1202	No
University Washington Physicians	Seattle, WA	181	1199	Yes
Mercy Springfield	Springfield, MO	349	1115	Yes
North Shore Long Island Jewish Syosset	Syosset, NY	259	1044	Yes
Carolinas Primary Care	Loris, SC	236	1024	Yes
Aurora Medical Group	Sheboygan, WI	206	1013	Yes
Novant Medical Group	Winston-Salem, NC	245	923	No

The “Challenge Data Set of Marshfield Physician Names” was used to evaluate the performance of a fuzzy match search engine. In the first comparison, the accuracy of fuzzy-match with Soundex algorithm was compared. Table 3 illustrates the results of this experiment. It should be emphasized that the current search engine returned “no result” for these 68 search terms. In the second evaluation, a comparison was done using simple Levenshtein distance in fuzzy-match versus customized Levenshtein distance. For similarity-based search methods, more than one result could be returned. As such, we also compared the efficiency of returning top ten matches versus top one match. The results suggest the top-one match already significantly outperforms Soundex, and the top-ten matches can further improve the retrieval performance.

**Table 3:** Comparing accuracy of Soundex, Levenshtein Distance (LD), and Fuzzy-Match on the Challenge Data Set of Marshfield Physician Names (N=68).

Search Engines	# Found	Percentage
Default Search Engine	0	0%
Soundex	16	24%
Fuzzy-match with simple LD (top one)	40	59%
Fuzzy-match with customized LD (top one)	48	71%
Fuzzy-match with simple LD (top ten matches)	52	77%
Fuzzy-match with customized LD (top ten matches)	61	90%

##  Discussion

### Principal Findings

This study focuses on the search engine used by patients to search the physician directory at a provider’s website. The same methods can be used to search any name directory system; for example, a directory of professors and staff members in the school of art and science of a university. It can also be used for Intranet searches. Staff members at Marshfield Clinic relate anecdotes about the inability to find the pager number for a physician in the Intranet directory, because they could not get the first character of the name spelled correctly. For example, “Przybylinski” (pronounced as “Shibilinski”) cannot be found under the directory using the starting letter “S”; however, using the fuzzy search engine presented in this paper, a top match can be found. The “Challenge Data Set of Marshfield Physician Names”, although small, can also be used in the future as a benchmark data set to test search engines of physician names.

### Conclusions

We designed and evaluated a fuzzy-match search engine for physician directories. The open-source code is available at Codeplex web site [13] and a reference implementation is demonstrated at datamarsh website under FuzzyMatch [14].
